# Lingual infarction in Wegener's Granulomatosis: A case report and review of the literature

**DOI:** 10.1186/1746-160X-4-19

**Published:** 2008-08-21

**Authors:** Lachlan M Carter, Eitan Brizman

**Affiliations:** 1 Specialist Registrar, Maxillofacial Surgery, Leeds Dental Institute, Clarendon Way, Leeds, LS2 9LU, UK; 2 Senior House Officer, Maxillofacial Surgery, Leeds Dental Institute, Clarendon Way, Leeds, LS2 9LU, UK

## Abstract

Wegener's granulomatosis (WG) is a multi-system disease, characterised by the triad of necrotising granulomata affecting the upper and lower respiratory tracts, disseminated vasculitis and glomerulonephritis. Oral lesions are associated with up to 50% of cases, although are rare as a presenting feature. The most common oral lesions associated with WG are ulceration and strawberry gingivitis. We review the literature regarding oral manifestations of WG and present a case of lingual infarction, an extremely rare oral lesion associated with WG, in a severe, rapidly progressive and ultimately fatal form of the disease.

## Background

Wegener's Granulomatosis (WG) is a multi-system disease, characterised by the triad of necrotising granulomata affecting the upper and lower respiratory tracts, disseminated vasculitis and glomerulonephritis. WG is included in the ANCA-associated small-vessel vasculitis group (including also microscopic polyangiitis, Churg-Strauss syndrome and renal-limited vasculitis). Oral lesions are associated with up to 50% of cases, although are rare as a presenting feature. The most common oral lesions associated with WG are ulceration and strawberry gingivitis. We present a case of lingual infarction, an extremely rare oral lesion associated with WG, in a severe, rapidly progressive and ultimately fatal form of the disease.

## Case Report

**Figure 1:**
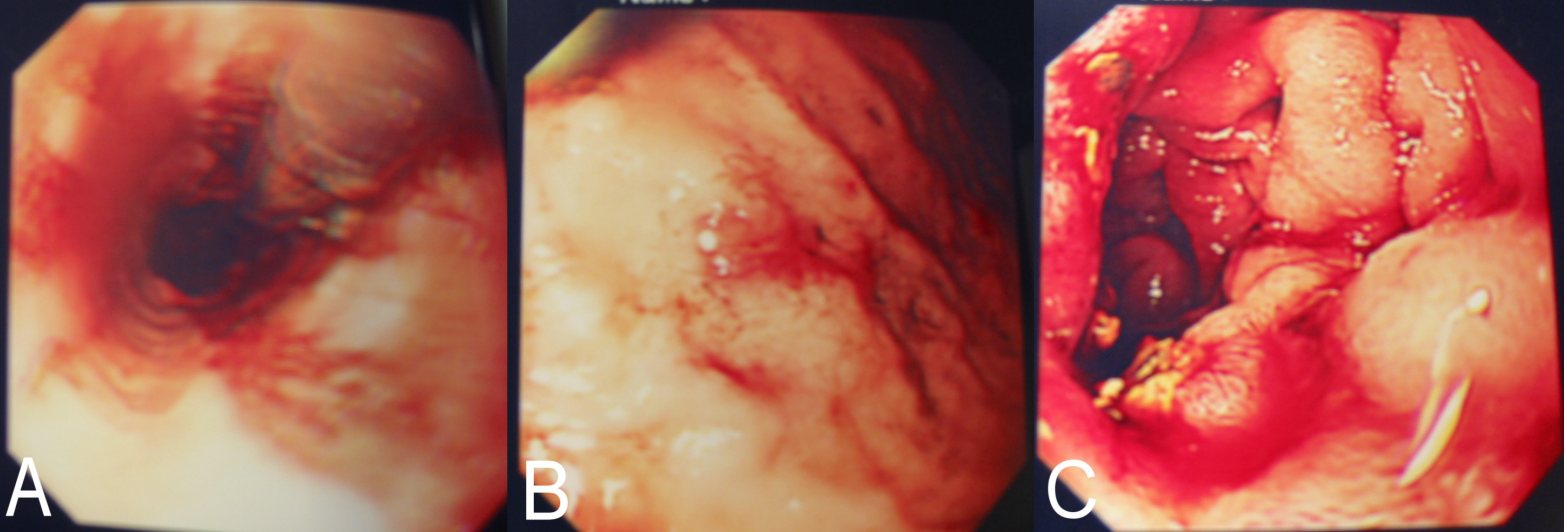
Endoscopic views of gastrointestinal lesions consistent with vasculitis: A oesophagus, B gastric mucosa, C duodenal mucosa.

**Figure 2:**
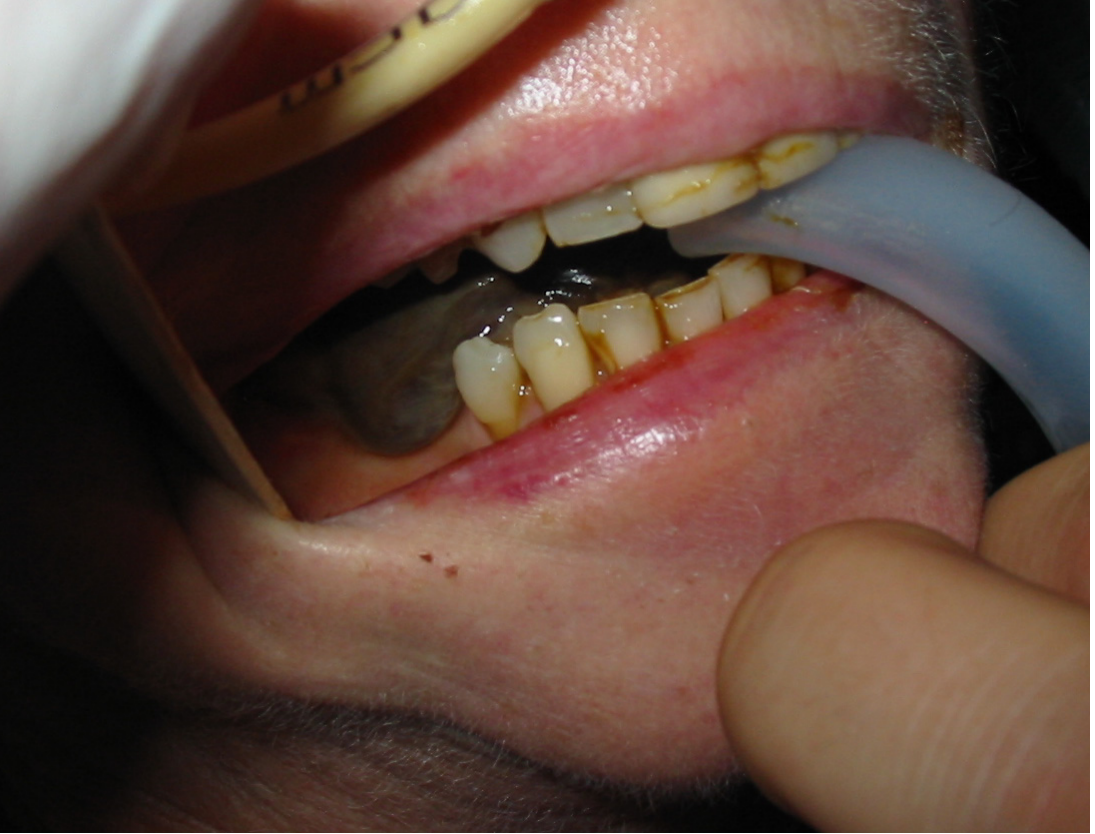
**Sloughing of the lingual mucosa**.

**Figure 3:**
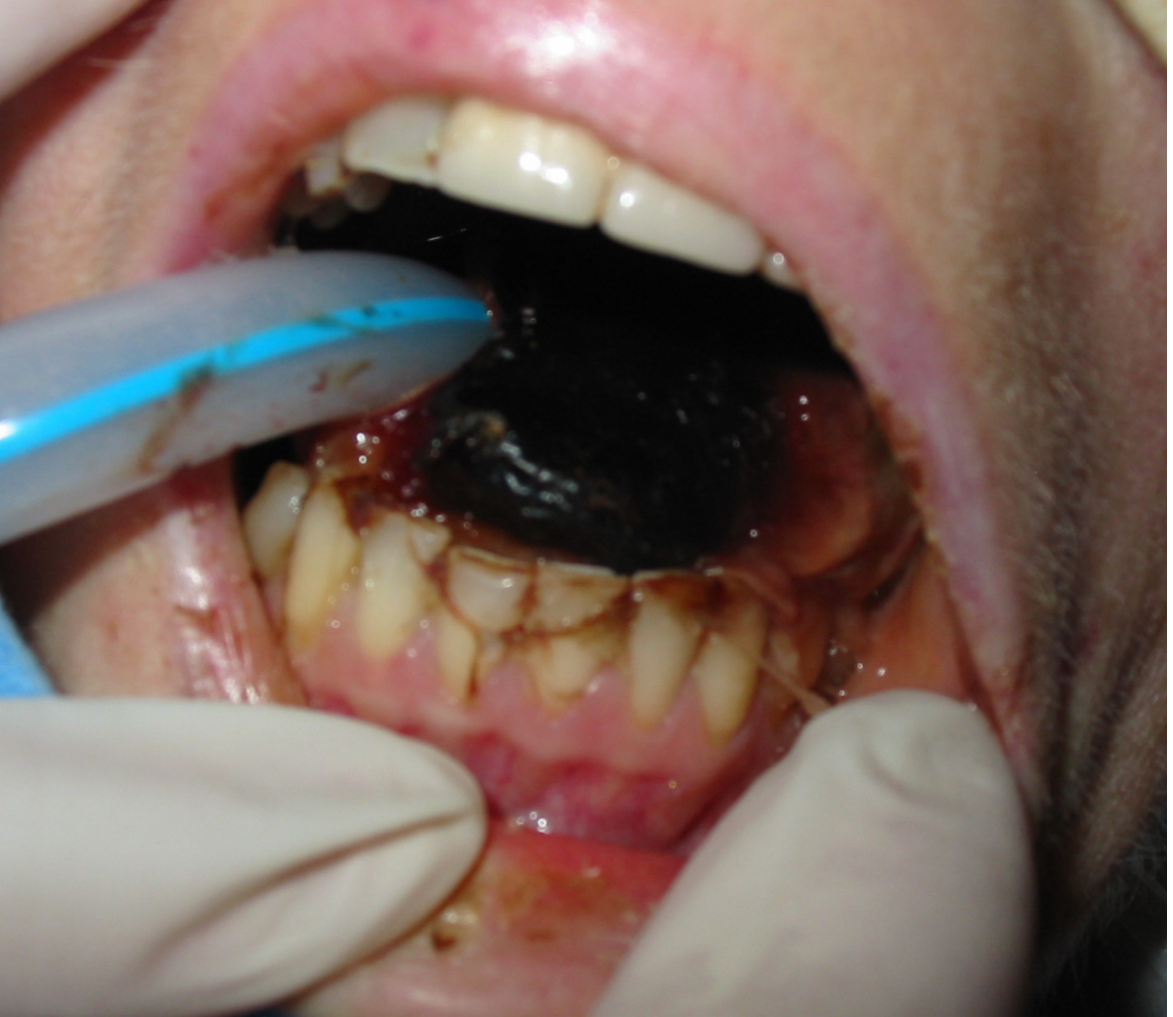
**Necrosis of anterior two thirds of tongue**.

A 56 year old female presented with headache, sinus pain, shortness of breath, cough productive of green sputum and haemoptysis. She had a history of bronchiectasis (diagnosed at age 20), hypertension, and a three month history of sinus problems with associated bilateral hearing loss. Chest radiograph revealed bilateral pleural effusions with apical opacities. Blood investigations revealed a C-reactive protein (CRP) of 454 mg/L and a creatinine of 76 umol/L. An initial diagnosis of lower respiratory tract infection was made and treatment with intravenous amoxicillin and erythromycin started. Pseudomonas was cultured from sputum after seven days, at which point intravenous gentamicin was started. She then developed pulmonary oedema and the haemoptysis worsened. Renal impairment also developed (urine protein/creatinine index 14395, urine protein 5.47 g/L, urine creatinine 3.8 mmol/L) and subsequently the gentamicin therapy was stopped. Her respiratory and renal function continued to deteriorate and she developed anterior t-wave inversion on ECG. Pulmonary haemorrhage secondary to systemic vasculitis was suspected. Blood investigations revealed haemoglobin (Hb) 9.0 g/dL, white cell count (WCC) 22.15 10^9^/L, platelets (PLT) 522 10^9^/L, CRP 227 mg/L, creatinine 159 umol/L, and positive cytoplasmic pattern anti-neutrophil cytoplasmic antibodies (cANCA) (ANCA protease-3 (PR3) 18 u/ml and ANCA myeloperoxidase (MPO) 1 u/ml). A diagnosis of Wegener's granulomatosis was considered most likely and intravenous methylprednisolone commenced. No upper airway lesions were identified on nasal endoscopy so no tissue biopsy could be taken. As a result of worsening respiratory and renal function she was intubated, ventilated and inotropic support with noradrenaline started. Nasogastric feeding was also commenced and plasmaphoresis undertaken. A renal biopsy was planned but she became progressively anaemic Hb 5.7 g/dL, thrombocytopenic PLT 35 10^9^/L, and her liver function deteriorated with a prothombin time of 31 s, making renal biopsy unsafe in the presence of coagulopathy. Gastrointestinal haemorrhage was suspected as the cause of anaemia. Oesophago-gastro-duodenoscopy (OGD) was performed which revealed oesophagitis, gastritis and duodenitis consistent with vasculitis, see figure 1. These lesions were injected with epinephrine and intravenous omeprazole was commenced. At this point, 19 days after initial presentation, sloughing of her lingual mucosa was noted, see figure 2. The mucosal sloughing involved the entire anterior two thirds of her tongue bilaterally. After the addition of intravenous cyclophosphamide her respiratory and renal function stabilised. The lingual sloughing persisted, and over the next 14 days progressed to an area of well demarcated necrosis of the anterior two thirds of the tongue bilaterally, see figure 3. The necrotic area began to separate but unfortunately she developed further pulmonary haemorrhage and her renal and cardiac function continued to deteriorate despite plasmaphoresis and inotropic support. Her condition was deemed irretrievable and supportive care was withdrawn. She died 48 days after initial presentation with the cause of death reported as multi-organ dysfunction syndrome (MODS) secondary to Wegener's granulomatosis. No post mortem examination was performed.

## Discussion

WG was first described by Freidrich Wegener in 1936 and 1939 [1,2]. In 1954, Godman and Churg further delineated the clinical and pathological features by describing the classical triad of necrotising granulomata affecting the upper and lower respiratory tracts, disseminated vasculitis and glomerulonephritis [3]. The aetiology of WG remains unknown.

In Europe the prevalence of WG is 5 cases per 100,000 population. The incidence is greater in Northern Europe. WG can occur in all racial groups, but predominantly affects Caucasians. Both sexes are affected equally. WG affects a wide age range (8–99 years) with a mean age at diagnosis of 40 years [4]. Historically WG, if untreated, had a poor prognosis with a mean survival time of 5 months [5]. In recent decades prognosis has improved with the introduction of immunosuppressive therapy using glucocorticosteroids and cyclophosphamide [6].

WG can be rapidly progressive or mild and indolent. Generalised symptoms such as fever, weight loss, fatigue and malaise may be present. Specifically, pulmonary manifestations such as cough, haemoptysis and pleuritis are the most common presenting symptoms. Renal disease can be the presenting feature in up to 18% of patients [6]. Renal involvement is characterised by abnormal renal function with red cell casts in urinalysis, and glomerulonephritis on renal biopsy. Renal involvement and a late age at onset are associated with an increased risk of mortality [7]. 80–90% of patients will have renal and/or pulmonary involvement at some time in their disease process.

WG can also affect the eyes, skin, joints, nervous system, ear, nose and throat [8]. The most common anatomical site for presenting lesions of WG is the upper airway. Up to 30% of patients may present with nasal problems including nasal obstruction, ulceration, septal perforation, mucus discharge, or epistaxis [9].

Diagnosis of WG is based on a combination of clinical, histological, biochemical and immunological features. Inconsistency of histopathological findings can make diagnosis based on this method in isolation difficult. In 1985 diagnosis was aided by the description of anti-neutrophil cytoplasmic antibodies (ANCA) associated with WG [10]. Two forms exist: cytoplasmic (cANCA) of which the principal target is protease-3 (PR3) and perinuclear (pANCA) which is directed against myeloperoxidase (MPO). These targets are antigens stored in the azurophilic granules of neutrophils and monocytes.

Lesions of the oral mucosa occur in 6–50% of patients with WG [11,12]. Duna et al reported oral lesions in 6–13% of patients but as the presenting feature in only 2% [9]. Oral and oropharyngeal ulcers resembling large aphthous ulcers are the most common oral lesion. Indeed at autopsy nearly all patients were reported to have oropharyngeal ulceration [1,13]. Hyperplastic gingivitis, dark-red to purple in colour with a granular surface, resembling an over-ripe strawberry, either generalised or affecting a single dental papilla, can be considered specific to WG [14-21]. In fact, the combination of 'strawberry gingivitis' exhibiting pseudoepitheliomatous hyperplasia, microabscesses and multi-nucleate giant cells upon biopsy with severe systemic upset can be considered diagnostic for WG [15]. Oro-antral fistulae [22], palatal osteonecrosis and labial mucosal nodules have also been reported [23]. WG can also affect the salivary glands with reported cases affecting the parotid, submandibular and sublingual salivary glands [24-26].

Lingual necrosis is a rare oral manifestation with only two previously reported cases. Bachmeyer et al reported a case of necrotic lingual ulceration which resolved with immunosuppressive therapy [23]. Rodgers et al reported a case of bilateral infarction of the tongue associated with a severe and rapidly progressive form of WG in 1992 [27]. The patient died 39 days after onset of symptoms (18 days after presentation). At post mortem examination the anterior two thirds of the tongue were infarcted. Our case was also fatal, exhibiting a similar clinical course with a similar anatomical distribution of lingual infarction. WG affects the small arteries of the lung and kidney causing pulmonary and renal infarction [20,27]. In our case the lingual arteries may have been similarly affected but unfortunately post mortem examination was not performed, therefore we cannot confirm that the lingual infarction was solely a result of lingual end-arteritis.

Lingual infarction can occur secondary to embolism, radiotherapy [28], tumour infiltration, radical neck dissection [29], transient ischaemic attack [30], and cardiac arrest [31]. Lingual infarction has also been reported in cranial arteritis, giant cell arteritis [32-35], and microscopic polyangiitis (MPA) [36]. WG and MPA share many similar clinical and histological features. Oral, upper airway, pulmonary and renal vasculitis are present in both conditions, however the vasculitis associated with WG is granulomatous whereas MPA exhibits non-granulomatous vasculitis [36].

Most patients with Wegener's granulomatosis exhibit cANCA with PR3 specificity and 25% exhibit pANCA with MPO specificity. In contrast microscopic polyangiitis exhibits cANCA with PR3 specificity in approximately 30% of patients and pANCA with MPO specificity in 60% of patients [36,37]. A positive ANCA result suggests a systemic vasculitis but in the absence of a tissue biopsy, cANCA/pANCA distribution may suggest either WG or MPA but cannot definitively differentiate between the two diagnoses. In our case a tissue biopsy could not be performed safely due to persistent thrombocytopenia and a post mortem examination was not performed. Therefore the presence of granulomatous vasculitis was not confirmed histologically but the histological differential diagnosis can be complicated in that not all biopsy material associated with WG exhibits the classical pathological triad of granulomatous infiltration, necrosis and vasculitis [38]. The clinical, biochemical and immunological features of our case were suggestive of a WG diagnosis.

Early diagnosis is important, expediting aggressive immunosuppressive therapy with glucocorticosteroids and cyclophosphamide, which can potentially limit a more severe systemic disease progression. Other treatment options include the use of trimethoprim and sulfamethoxazole either as a stand alone treatment or in combination with glucocorticosteroids and cyclophosphamide [9]. More recent treatment options include Cyclosporin, intravenous pooled immunoglobulin, anti-CD20 monoclonal antibodies (Rituximab) and anti-tumour necrosis factor alpha with the latter two options restricted to refractory and relapsed disease [39,40]. Regular review and maintenance therapy are also important to identify and prevent relapse. A multidisciplinary approach must be undertaken involving oral and maxillofacial surgeons, oral physicians, otorhinolaryngologists, rheumatologists, renal and respiratory physicians, ophthalmologists, and ITU supportive care if required. WG should be considered in the presence of oral lesions associated with a systemic illness. An oral biopsy and blood investigations assessing full blood count, renal and hepatic function, inflammatory markers and autoimmune status, specifically cANCA and pANCA, should be performed.

Oral lesions are associated with the onset of active systemic disease [12]. Therefore isolated oral lesions may herald the onset of further systemic involvement. Mahr et al reported that oral, ear, nose or throat involvement was not associated with survival in their multivariate analysis. Mahr et al did indicate that granulomatous WG probably has a more benign course than vasculitic WG [41]. Our case and the previously reported case of lingual infarction were associated with a severe, rapidly progressive and ultimately fatal form of WG. Thus lingual infarction as a result of vasculitis may indicate more aggressive disease. The severity of oral mucosal lesions, even in the absence of systemic signs, may therefore reflect or predict the severity of the generalised systemic disease and indicate a more aggressive vasculitis.

## Competing interests

The authors have no financial and personal relationships with other people, or organisations, that could inappropriately influence (bias) their work, all within 3 years of beginning the work submitted.

## Authors' contributions

LMC and EB prepared the case report, discussion and manuscript. Both authors read and approved the final manuscript.

## Consent

Written consent for publication of the clinical images could not be obtained because the patient died before written consent could be recorded.

## References

[B1] Wegener F (1936). Uber generalisierte, septische Gefaesserkrankungen. Verh Dtsch Ges Pathol.

[B2] Wegener F (1939). Uber eine eigenartige Rhinogene Granulomatose mit besondere Beteilgung des Arteriensystems and der Nieren. Beitrage Pathologie Anatomie.

[B3] Godman GC, Churg J (1954). Wegener's granulomatosis: pathology and review of the literature. Arch Pathol Lab Med.

[B4] Gubbels SP, Barkhuizen A, Hwang PH (2003). Head and Neck manifestations of Wegener's granulomatosis. Otolaryngol Clin North Am.

[B5] Walton EW (1958). Giant-cell granuloma of the respiratory tract (Wegener's granulomatosis). Br Med J.

[B6] Fauci AS, Haynes BF, Katz P (1983). Wegener's granulomatosis: prospective clinical and therapeutic experience with 85 patients for 21 years. Ann Intern Med.

[B7] Aasarod K, Iverson BM, Hammerstrom J, Bostad L, Vatten L, Jorstad S (2000). Wegener's granulomatosis: clinical course in 108 patients with renal involvement. Nephrol Dial Transplant.

[B8] De Remee RA, McDonald TJ, Harrison EJJ, Coles DT (1976). Wegener's Granulomatosis, Anatomic Correlates, A Proposed Classification. Mayo Clin Proc.

[B9] Duna GF, Galperin C, Hoffman GS (1995). Wegener's granulomatosis. Rheum Dis Clin North Am.

[B10] Van der Woude FJ, Rasmussen N, Lobatto S (1985). Autoantibodies against neutrophils and monocytes: tool for diagnosis and marker of disease activity in Wegener's granulomatosis. Lancet.

[B11] Patten SF, Tomeki KJ (1993). Wegener's granulomatosis: Cutaneous and oral mucosal disease. J Am Acad Dermatol.

[B12] Frances C, Du LT, Piette JC, Saada V, al (1994). Wegener's granulomatosis.  Dermatological manefestations in 75 cases with clinicopathologic correlation. Arch Dermatol.

[B13] Wegener F, Staemmler M (1967). Die pneumogene allgemeine Granulomatose (PG) - sog.  Wegnersche Granulomatose.. Lehrbuch der speziellen pathologischen Anatomie, Ergaenzungsbans, I/1.

[B14] Lilly J, Todd J, Lew D, Vincent S, Lilly G (1998). Wegener's granulomatosis presenting as oral lesions.  A case report. Oral Surg Oral Med Oral Pathol Oral Radiol Endod.

[B15] Napier SS, Allen JA, Irwin CR, McCluskey DR (1993). Strawberry gums: a clinicopathological manifestation diagnositc of Wegener's granulomatosis?. J Clin Pathol.

[B16] Allen CM, Camisa C, Saleweski C, Weiland JE (1991). Wegener's granulomatiosis: report of three cases with oral lesions. J Oral Maxillofac Surg.

[B17] Eufinger H, Machtens E, Akuamoa-Boateng E (1992). Oral manifestations of Wegener's granulomatosis.  Review of the literature and report of a case. Int J Oral Maxillofac Surg.

[B18] Glass EG, Lawton LR, Truelove EL (1990). Oral presentation of Wegener's granulomatosis. JADA.

[B19] Cohen PS, Metzler JA (1981). Strawberry gums.  A sign of Wegener's granulomatosis. JAMA.

[B20] Raustia AM, Autio-Harmainen HI, Knuuttila MLE, Raustia JM (1985). Ultrastuctural findings and clinical follow-up of 'strawberry gums' in Wegener's granulomatosis. J Oral Pathol.

[B21] Scott J, Finch LD (1972). Wegener's granulomatosis presenting as gingivitis. oral Surg Oral Med Oral Pathol.

[B22] Edwards MB, Buckerfield JP (1978). Wegener's granulomatosis: A case with primary mucocutaneous lesions. Oral Surg Oral Med Oral Pathol.

[B23] Bachmeyer B, Petitjean B, Testart F, Richecoeur J, Ammouri W, Blum L (2006). Lingual necrosis as the presenting sign of Wegener's granulomatosis. Clin Exp Dermatol.

[B24] Berge S, Niederhagen B, von Lindern JJ, Appel T, Reich RH (2000). Salivary gland involvement as an initial presentation of Wegener's disease.  A case report. Int J Oral Maxillofac Surg.

[B25] Lustmann J, Segal N, Markitziu A (1994). Salivary gland involvement in Wegener's granulomatosis.  A case report and review of the literature. Oral Surg Oral Med Oral Pathol Oral Radiol Endod.

[B26] Crean SJ, Adams R, Bennett J (2002). Sublingual gland involvement in systemic Wegener's granulomatosis: a case report. Int J Oral Maxillofac Surg.

[B27] Rodgers H, Quirke P, Lipkin GW, Brownjohn AM (1992). Infarction of the tongue in Wegener's granulomatosis. Br J Clin Pract.

[B28] Miller SH, Rudolph R (1990). Healing in the irradiated wound. Clin Plast Surg.

[B29] Gault DT (1988). Tongue necrosis after radical neck dissection. Head Neck Surg.

[B30] Orita Y, Ogawara T, Yorizane S, Nannba Y, Akagi H, Nishizaki K (2000). Necrosis of the tongue after transient ischaemic attack. Oral Surg Oral Med Oral Pathol Oral Radiol Endod.

[B31] Libersa P, Loison-Blanchard C, Nawrocki L, Duquesnoy S (2002). Bilateral necrosis of the tongue consecutive to cardiac arrest. J Oral Maxillofac Surg.

[B32] McRorie ER, Chalmers J, Campbell IW (1994). Lingual infarction in cranial arteritis. Br J Clin Pract.

[B33] Crevitis I, Hermans R, Wilms G, Baert AL (1996). Tongue necrosis as a complication of temporal arteritis: CT and angiographic findings. J Belg Radiol.

[B34] Biebl MO, Hugl B, Posch L, al (2004). Subtotal tongue necrosis in delayed diagnosed giant cell arteritis: a case report. Am J Otolaryngol.

[B35] Ciantar M, Adlam DM (2007). Glossodynia and necrosis of the tongue caused by giant cell arteritis. Br J Oral Maxillofac Surg.

[B36] Shiboski CH, Regezi JA, Sanchez HC, Silverman SJ (2002). Oral lesions as the first clinical sign of microscopic polangiitis: a case report. Oral Surg Oral Med Oral Pathol Oral Radiol Endod.

[B37] Savige J, Gillis D, Benson E, Davies D, Esnault V, Falk RJ, al (1999). International concensus statement on testing and reporting of antineutrophil cytoplasmic antibodies. Am J Clin Pathol.

[B38] Devaney KO, Travis WD, Hoffman G, Leavitt R, R. L, Fauci AS (1990). Interpretation of Head and Neck Biopsies in Wegener's Granulomatosis. Am J Surg Pathol.

[B39] Allen NB, Caldwell DS, Rice JR (1993). Cyclosporin A therapy for Wegener's granulomatosis. Adv Exp Med Biol.

[B40] Jayne DR, Lockwood CM (1993). Pooled intravenous immunoglobulin in the management of systemic vasculitis. Adv Exp Med Biol.

[B41] Mahr A, Girard R, Agher R, Guillevin L (2001). Analysis of factors predictive of survival based on 49 patients with systematic Wegener's granulomatosis and prospective follow-up. Rheumatology.

